# A potential role of transposon IS431 in the loss of *mecA* gene

**DOI:** 10.1038/srep41237

**Published:** 2017-01-25

**Authors:** Aihua Wang, Kai Zhou, Yang Liu, Liang Yang, Qin Zhang, Jing Guan, Nanshan Zhong, Chao Zhuo

**Affiliations:** 1 State Key Laboratory of Respiratory Diseases, the first affiliated hospital of Guangzhou Medical College, Guangzhou, China; 2 State Key Laboratory for Diagnosis and Treatment of Infectious Diseases; Collaborative Innovation Center for Diagnosis and Treatment of Infectious Diseases, the First Affiliated Hospital of Medicine School, Zhejiang University, Hangzhou, China; 3 National University of Singapore, Singapore; 4 Daxian people’s hospital, Dazhou, China

## Abstract

Acquisition of a vancomycin-resistance-determinant may trigger deletion of the *mecA* gene. However, the molecular mechanisms involved remain largely unknown. In this study, we successfully produced vancomycin-intermediate-resistant *Staphylococcus aureus* (VISA) from Methicillin-resistant-*S. aureus* (MRSA) through serial passages with vancomycin. Five MRSA isolates achieved a vancomycin MIC of >8 mg/ml after 45-day serial exposure to vancomycin. After 20-day passages in media without antibiotics, three of the isolates were restored to pre-induction levels, whilst the remaining 2 (3503-1 and 4126-1) retained a vancomycin MIC >6 mg/ml. The oxacillin MICs for strain 3503-1 and its induced equivalents 3503VR6 and 3503VR10, were 512 μg/ml, <2 μg/ml, and <2 μg/ml, respectively. Oxacillin MICs for 4126-1 and its induced strain 4126VR10 were 512 μg/ml and 128 μg/ml, respectively. Strains 3503-1 and 3503VR6 were sensitive to gentamicin while 4126-1 and 4126VR10 were resistant. PFGE analysis demonstrated that comparing to the parental strain 3503VR6 and 3503VR10 lacked a DNA fragment of 40-kb and 80-kb, respectively. Both deleted regions localized around the transposon IS*431*. The deletion region of 3503VR10 was further investigated by whole-genome sequencing. We conclude that transition from MRSA to VISA may cause deletion of the mobile genetic element staphylococcal cassette chromosome *mec* (SCC*mec*), and possibly be mediated by IS*431*, resulting in increased susceptibility to oxacillin.

Methicillin-resistant *Staphylococcus aureus* (MRSA) is a hospital-acquired pathogen of great concern globally. Along with AIDS and viral hepatitis B, MRSA infection is regarded as one of three major infectious diseases threatening human health^1^. Vancomycin is currently used to treat MRSA infections, but the emergence of vancomycin-intermediate*S. aureus*(VISA), heterogeneous vancomycin-intermediate*S. aureus*(hVISA), and high-level vancomycin-resistant*S. aureus*(VRSA), is a cause for concern. Although it is known that the transitioning of vancomycin susceptible MRSA (VSSA) to VISA is linked to changes in DNA sequences and transcriptional regulations, the exact mechanism involved remains largely unknown.

The emergence of VISA from a previously vancomycin susceptible bacterial strain may be triggered either by exposure to vancomycin or other antibiotics, or when *murF* or *pbpB* is inactivated, resulting in up-regulated expression of relevant stimulatory factors such as the VrsSR two-component system, which subsequently affect cell wall biosynthesis^2^,^3^. Notably, reduced susceptibility to glycopeptide class of antibiotics and increased susceptibility to β-lactam antibiotics, has been observed during the transition of VISA from VSSA, a phenomenon referred to as the ‘see-saw effect’. However, the underlying mechanism of this phenomenon remains poorly understood^4^.

In this study, we report on five MRSA strains in which susceptibility to oxacillin decreased during transformation from vancomycin susceptible MRSA to VISA *in vitro*. In the most extreme case, the oxacillin minimum inhibitory concentration (MIC) for one strain decreased from 512 μg/ml to 1 μg/ml. Furthermore, the *mecA* gene was deleted in this particular strain, suggesting that loss of this gene may be involved in the acquisition of vancomycin resistance. However, it is plausible that other unknown mechanisms are involved. Induced strains were characterized genotypically and compared to parental strains.

## Results

### Bacterial strains

Details of all the bacterial strains used in this study are shown in Table 1.

### Vancomycin induction

**Figure 1:**
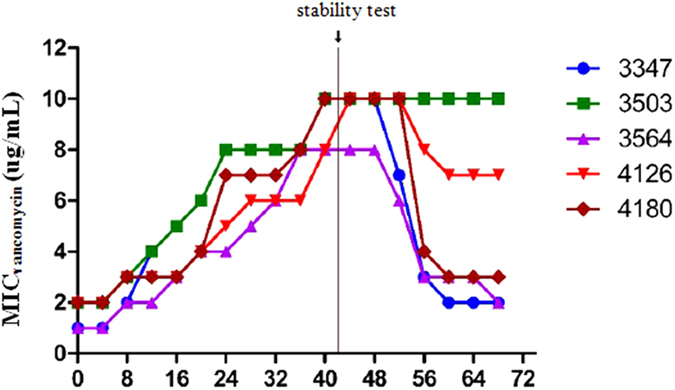
Process of induction of VISA from MRSA.

**Figure 2:**
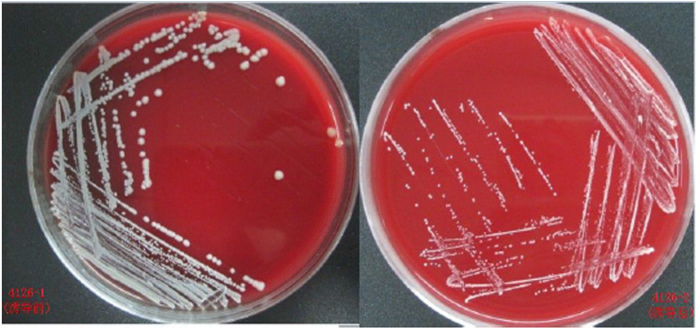
VISA colony morphology.

Five MRSA strains were passaged through several low vancomycin concentrations. The vancomycin MICs for strains 3503-1, 3347, 3564, 4126-1 and 4180, all increased following induction by a series of increasing vancomycin concentrations (0.375–12 μg/ml) for 45 days, reaching 6 μg/ml by day 30 and the maximal value of 10 μg/ml by day 45 (Fig. 1). In order to ascertain the permanency of the newly acquired MICs, the induced strains were continuously passaged for 20 days on plates without any antibiotics. The vancomycin MICs of three strains (3347, 3564 and 4180) were restored to the pre-induced levels (2–3 μg/ml), whilst the other two strains (3503-1 and 4126-1) retained the acquired vancomycin MICs of 10 and 7 μg/ml, respectively. Specifically for strain 3503-1, two induced strains with vancomycin MIC of 6 μg/mL (identified as 3506V6), and MIC of 10 μg/mL (identified as 3506V10), produced after 20 and 45 days of induction, respectively, were further tested. Interestingly, the colonies of induced strains were smaller and drier than their parental strains (Fig. 2). The oxacillin MICs of all the five strains decreased by between 4- to 512-fold during vancomycin induction (data not shown). In particular, the oxacillin MIC of strain 3503-1 decreased from 512 μg/ml to 1 μg/ml during the 45-day passage, and was shown to have lost the *mecA* gene by PCR confirmation (see below). All the strains, except 3503-1 and its induced equivalents, were resistant to gentamicin. Further details on the drug susceptibilities of all the strains used in this study are shown in Table 1.

### PFGE analysis of DNA fragment patterns

**Figure 3:**
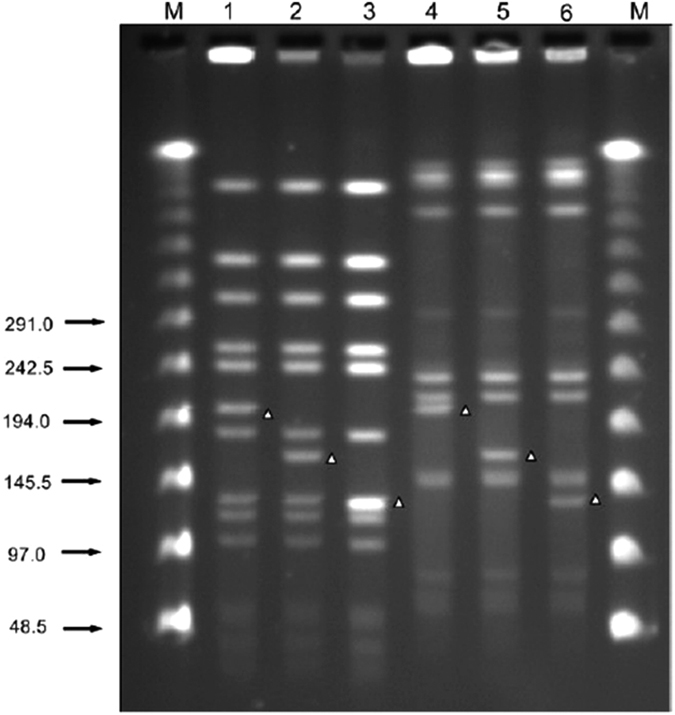
PFGE analysis of strain 3503-1 and their induced strains. The PFGE DNA ladder is shown. Lanes 1 and 4 = strain 3503-1. Lanes 2 and 5 = strain 3503VR6. Lanes 4 and 6 = strain 3503VR10. Lanes 1–3 = DNA digested by SmaI. Lanes 3–6 = DNA digested by RsrII.

To gain further insights into the homology of parental and induced strains, the five MRSA strains and their induced VISA strains were further analyzed by PFGE. Each of the four strains (3347, 3564, 4126 and 4180) had a unique DNA fragment pattern which was similar to its induced equivalent. In contrast, strain 3503-1 had a different DNA fragment pattern compared to its induced equivalent strains 3503VR6 and 3503VR10 (Fig. 3). Restriction endonuclease digestion with *SmaI* revealed a distinct fragment at 200 kb for the four strains with similar DNA fragment pattern, but this fragment was shifted to 160 kb in strain 3503VR6. However, for strain 3503VR10, there was no band at 160 kb position, but instead there was one at 120 kb which was much brighter than that in 3503-1 and 3503VR6. Pattern differences were even more significantly shown by the PFGE of *RsrII-*digested DNA. Taken together, these results indicate that 40 kb and 80 kb fragments were deleted from strains 3503VR6 and 3503VR10, respectively.

### Confirmation of the staphylococcal cassette chromosome *mec* (SCC*mec*) deletion sites in strain 3503-1

**Figure 4:**
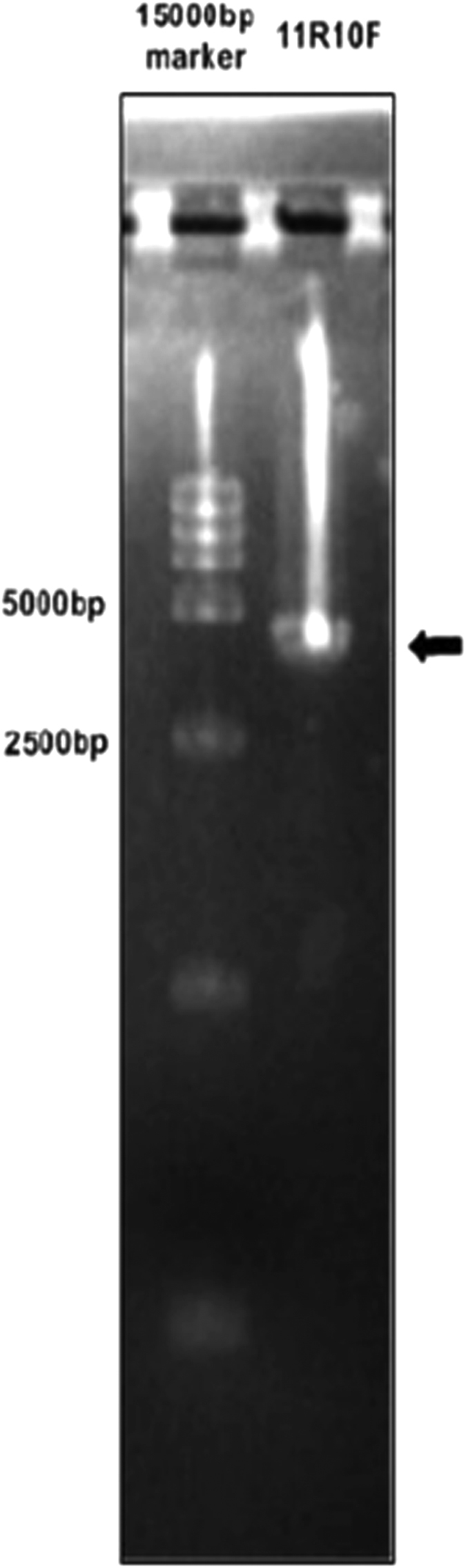
SCC*mec* conjunction PCR product of 3503VR6.

**Figure 5:**
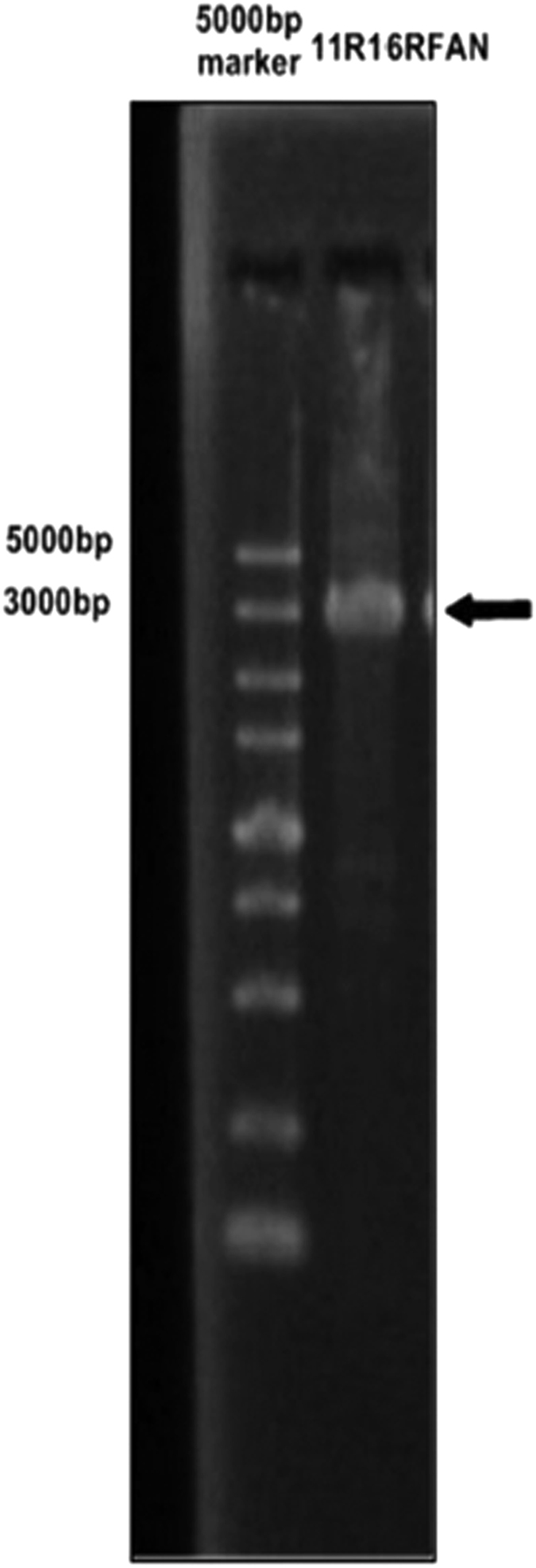
SCC*mec* conjunction PCR product of 3503VR10.

Based on the SCC*mec* gene sequence of strain MRSA N315 downloaded from GenBank, 18 previously published^5^ and newly designed, primer pairs were used to investigate the deleted regions of induced strains 3506VR6 and 3503VR10, with strain 3503-1 used as a positive control. A series of primers listed in Table 2 were used to amplify the target fragments. No PCR products were generated from strain 3503VR6 by five sets of primers (map2, map4, map7, map8 and map9), while PCR products could only be amplified from 3503VR10 by primers map10 and map16. Based on the gene map of fragments from ORFX to spa of reference strain MRSA N315, these results suggested that the SCC*mec* element deletion sites in 3503VR6 and 3503VR10 may be located between the binding sites of primers map11 and map10, and map11 and map16, respectively. A 4-kb fragment designated 11R10F was successfully amplified with primers map10F and map11R from strain 3503VR6 (Fig. 4). Likewise, another 4-kb fragment (11 R16RFAN) was amplified from strain 3503VR10 using primers map11R and map16RFAN (Fig. 5).

### SCC*mec* deletion mapping

**Figure 6:**
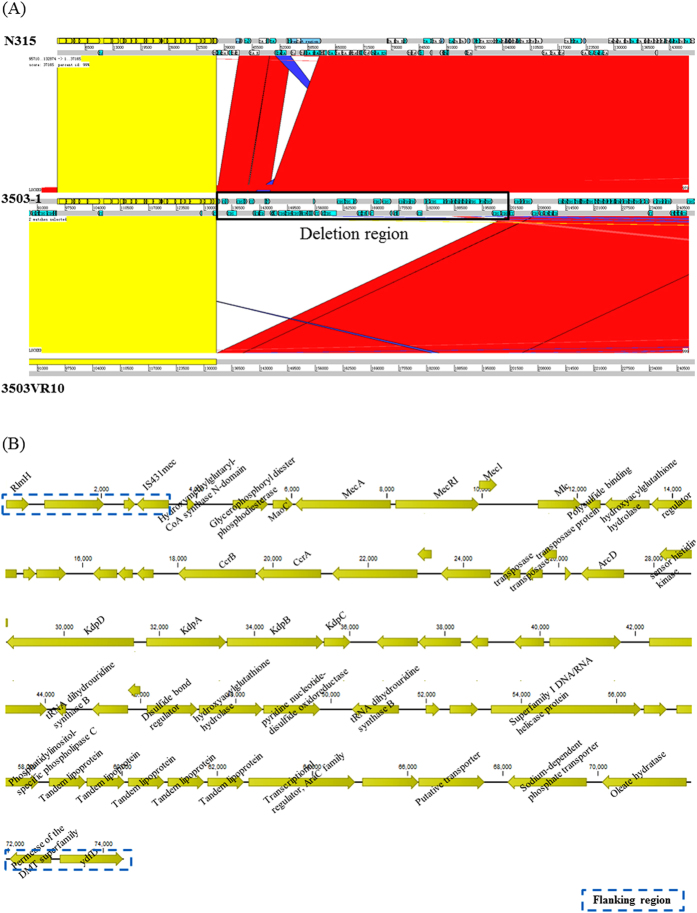
Syntany analysis of the region containing the SCC*mec*. (**A**) The region containing the SCC*mec* highlighted in the box was absent in 3503VR10. (**B**) Details of the region deleted in 3503VR10. Flanking regions are shown in box with dash lines.

The first 2-kb sequences (between fragments 11R10F and 11R16RFAN) of the deleted regions were identical to the reference sequence of MRSA N315 downloaded from the Genbank database. Deletion fragment 11R10F started at position 37,166 bp, which is 56 bp upstream of the gene SA0026 (IS431), and ended at position 81,168 bp which is within the *kdpC* gene, whereas fragment 11R16RFAN ended at position 116,954 bp, within gene SA0102 (Figure S1. SCC*mec* deletion site in 3503VR6 and 3503VR10). The deletions in strains 3503VR6 and 3503VR10 were 44,003 bp and 79,789 bp long, respectively. Both deleted regions included *ccrAB, Tn554*, and part of the *mecA* gene of the SCC*mec* region (Figure S2. Schematic diagram showing the deletion regions of strains 3503VR6 and 3503VR10). Furthermore, both deletions included SA0102 that encodes a 67-kDa myosin-cross-reactive streptococcal antigen. To confirm the results of deletion mapping, 3503-1 and 3503VR10 were sent to whole-genome sequencing (WGS). The deletion region was demonstrated by the syntany analysis (Fig. 6A). Details of the deletion region are shown in Fig. 6B. No further deletions were detected between 3503-1 and 3503VR10. Additionally, few Single-nucleotide polymorphisms (SNPs) were identified between 3503-1 and 3503VR10 (Table 3). Of note, one of SNPs located within *tagH* gene encoding a teichoic acid export ATP-binding protein.

### Single-nucleotide polymorphisms (SNPs) associated with vancomycin resistance in 4126-1

As no deletions were detected between 4126-1 and 4126VR10, we further inspected the potential mechanisms involved in the vancomycin resistance. These two strains were send to WGS, and in total of 11 high-quality SNPs were identified between them (Table 4). Notably, two non-synonymous SNPs resulting in nonsense mutations were detected in genes *murA* and *gad*, respectively. *murA* gene encodes UDP-N-acetylglucosamine 1-carboxyvinyltransferase catalyzing the first committed step on peptidoglycan synthesis, and *gad* gene encodes mannosyl-glycoprotein endo-beta-N-acetylglucosaminidase catalyzing the hydrolysis of the N,N’-diacetylchitobiose moiety of asparagine-linked oligosaccharides of various glycoproteins.

### Quantitative PCR analysis

**Figure 7:**
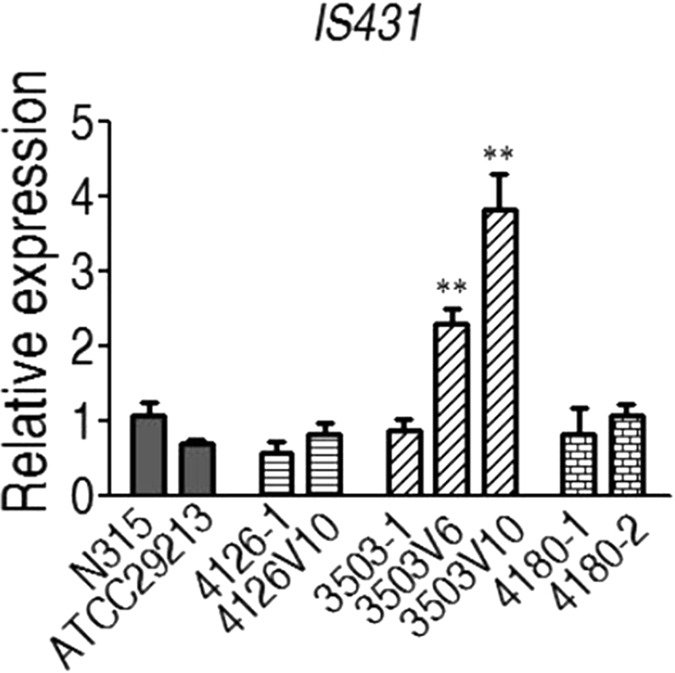
Relative quantification of *IS431* expression using 16 s rRNA genes for normalization under different experimental conditions.

To determine the role of IS*431* in the vancomycin-induced deletion of SCCmec, the expression level of IS*431* was compared between three strains (3503-1, 4126, 4180) and their associated induced variants using the 16 s rRNA gene as the reference (Fig. 7). IS*431* expression increased significantly in 3503V6 and 3503V10 comparing to their parent strain 3503-1 (*p* < 0.01), while no significant differences were observed for strains 4126 and 4180 and their induced equivalents.

## Discussion

It has been reported that MRSA is resistant to all β-lactam antibiotics due to acquisition of the SCC*mec* element from related organisms, possibly via horizontal gene transfer. However, the mechanism involved is still unclear. The stability of the SCC*mec* element in an organism may be affected by long-term storage and exposure to high temperatures, ultraviolet, and antibiotics. In a report by Sieradzki *et al*., an MRSA strain lost resistance to oxacillin after acquisition of vancomycin resistance. In this strain, *mecA* function was lost due to the insertion of a replicon^4^. Similarly, in the present study, *mecA* was not detected in VISA strains 3503VR6 or 3503VR10 by PCR, and PFGE and WGS confirmed that indeed *mecA* was deleted in both strains due to an endonuclease fragment jump at 190 kb (Fig. 3).

Deletion of the SCC*mec* cassette from VISA has been reported previously^6^,^7^. Specifically, Bhateja *et al*. found that *mecA* was deleted following induction of vancomycin resistance in one of eight VISA-induced strains^6^. Likewise, in the present study, only one out of 5 strains (3503-1) induced, lost the *mecA* gene.

Deletion of *mecA* is dependent on several factors. The type of SCC*mec* involved may be important; Wong *et al*. demonstrated that deletions were common in isolates harboring SCC*mecII*. However, in the present study, strain 4126-1, which like strain 3503-1 possessed the SCC*mecII* element, did not exhibit *mecA* gene deletion, suggesting that further studies involving more bacterial strains are needed before conclusions can be drawn.

Previous studies also indicate that deletion of *ccrA* and *ccrB* genes may also play a role in *mecA* deletion as reported by Adhikari *et al*. in a study involving 49 induced strains, some of which exhibited decreased resistance to oxacillin, and one of which had undergone *mecA* deletion^8^. Likewise, *ccrA* and *ccrB* was lost together with the SCC*mec* in strain 3503VR10 (Fig. 6).

Insertion of transposon IS*431* may also be responsible for *mecA* gene deletion. Findings from our study seem to confirm this as PCR analysis revealed that the 3′-end of SCC*mec* was deleted in strains 3503VR6 and 3503VR10, and that the deletion was 56-bp upstream of transposon *IS*431. This transposon which is ubiquitous in all SCC*mec* regions studied to date, is always located downstream of *mecA*, and may play an important role in the movement and stability of *mecA*^9^. *In vitro* VISA-induced strains may undergo deletion of part of SCC*mec* during insertion of transposon IS*431*, as observed by Reipert *et al*. who identified a VISA strain with a *mecA* deletion located downstream of transposon IS*431*^10^. In the present study, MRSA strains were successfully induced by vancomycin, and a vancomycin MIC of 6 μg/ml was associated with the deletion of a 40-kb fragment containing the SCC*mec* element. Further vancomycin induction resulted in the deletion of a further fragment upstream of SCC*mec*. These findings suggest that transposon IS*431* may not only involving in the deletion of SCC*mec* fragment, but also splice other fragments in the *Staphylococcus aureus* genome. Additionally, the higher expression of IS*431* detected in the 3503 induced variants (3503V6 and 3503V10) suggest that IS*431* may trigger the degradation function, resulting in loss of 40-kb fragment in 3503V6 and 80-kb in 3503V10, although the degradation mechanism remains unclear.

This study has some limitations. First, some phenotypic changes that occurred during the acquisition of vancomycin resistance cannot be directly linked to loss of SCC*mec*. Specifically, strain 3503-1 lost resistance to oxacillin upon acquiring vancomycin resistance (MIC of 6 μg/ml). However, this strain exhibited resistance to vancomycin prior to loss of the SCC*mec*-associated oxacillin resistance. It is thus difficult to directly attribute the loss of SCC*mec*-associated oxacillin resistance to the acquisition of vancomycin resistance from the existing data. We postulate that the loss of SCC*mec* (and associated methicillin resistance) is adaptive for bacteria acquiring vancomycin resistance. Additionally, a SNP was detected in *tagH* gene encoding a teichoic acid export ATP-binding protein, and it is known that changes of teichoic acid can affect the susceptibility to vancomycin in *S. aureus*^11^. Whether the mutation of *tagH* involved in the alteration of vacomycin susceptibility should be determined in the future.

Furthermore, we did not analyze methicillin -sensitive VISA at the MIC of 6 μg/ml, and this should be performed in the future. The loss of SCC*mec*-associated drug resistance was induced by two rounds of deletions, which to our knowledge is the first this has been reported. Although strains 4126 and 3503 were identical in the regions sequenced, their resistance to gentamicin was different, and we hypothesize that this may be SCC*mec*-associated. Second, despite confirming that *aac(6′)-aph(2w″)* was present in strain 4126, we did not determine whether this gene was localized chromosomally or on a plasmid, and thus further genetic analysis of these strains is needed. It has been reported that MRSA containing penicillin resistance plasmids may have increased *mecA* stability^12^, which needs further investigation.

An understanding of the mechanism by which bacterial strains with higher levels of vancomycin resistance lose resistance to methicillin/oxacillin is important for clinical diagnosis. Bacterial isolates resistant to vancomycin may affect drug resistance to oxacillin and even induce resistance in β-lactam-sensitive strains. Currently, the isolation rate of MRSA from clinical samples is much higher than that of MSSA, and β-lactam antibiotics are increasingly unsuitable for treatment of MRSA infections. The results presented here suggest that treating MRSA with vancomycin may ultimately lead to vancomycin resistance, but may also enhance or even restore susceptibility to methicillin/oxacillin, which suggests that there may be potential for effective combination therapies.

## Materials and Methods

### Bacterial strains

All bacterial strains used in this study are listed in Table 1. Strain N315, which is the MRSA prototype strain for SCC*mec* type II and positive for the HVR-IS*431*-pUB110-IS*431-dcsorfx* structure, was used as positive control. Strain Mu50, which is the referred strain for ST5-MRSA-II and positive for VISA. Details of the 45-day serial passage selection process have been previously reported^8^. Briefly, the strains were inoculated on blood agar plates, and single colonies were re-suspended in 0.9% NaCl solution to a McFarland standard of 10^5^ CFU/ml. After this, 10 μl of the suspension was inoculated on brain heart infusion (BHI) plates containing 0.375 μg/ml vancomycin and incubated overnight at 37 ^o^C. Vancomycin-adapted colonies were subsequently cultured on BHI agar plates (Oxoid, Basingstoke, England) containing 2, 4, 6, 8 and 12 μg/ml vancomycin. Vancomycin MIC values were determined every four days using the broth microdilution method. E-tests were used to confirm MICs ≥ 4 μg/ml during the serial passage.

For the stability assay, VISA strains were cultured on BHI agar plates without vancomycin once per two days, and the passage was continuous for 20 days.

### *In vitro* susceptibility assay

The broth microdilution method was used to determine MICs for vancomycin, oxacillin, and gentamicin^13^. Concentrations used for vancomycin MIC determination were 10, 8, 7, 6, 5, 4, 3, 2 and 1 μg/ml, and 512, 256, 128, 64, 32, 16, 8, 4 and 2 μg/ml, for oxacillin. Bacteria were cultured at 37 °C for 24 h, and *S. aureus* ATCC29213 was used as a control.

### SCCmec typing and mecA gene detection

The primers used for SCCmec typing and *mecA* gene detection were acquired from previous study^14^ and showed in Table 5. SCCmec PCR amplification reaction conditions: 94 °C denaturation for 5 mins, 94 °C denaturation for 45 s, 65 °C annealing for 45 s, 72 °C extension for 1.5 mins, the above steps for 10 cycles. Then 94 °C denaturation for 45 s, 55 °C annealing for 45 s, 72 °C extension for 1.5 mins, the above steps for 25 cycles, and finally 72 °C extending for 10 mins.

### PFGE analysis

Bacteria were re-suspended in 12 mM Tris buffer to a final concentration of 10^5^ CFU/ml. The resulting suspensions were mixed with 2% low melting agarose (1:1) to prepare mini gels that were subsequently incubated with 20 U of lysostaphin overnight, and digested with 40 U of protein kinase K for 16 h. DNA was digested by *SmaI* and *RsrII* for 6 h, and the resulting DNA fragments compared for parental and induced strains as previously reported^15^.

### Analysis of the region upstream and downstream of *mecA* gene

#### Primer design

Based on the SCC*mec* gene sequence of MRSA N315 downloaded from the GenBank (Accession number: D86934.2), primers were designed and synthesized (Table 2). Fourteen primer pairs were used to map the fragments from ORFX to spa as per reference strain MRSA N315. Primer pairs map2, map4, map7, map8, map9 were acquired from the previous study^16^, and the others were designed in this study using the primer walking strategy. The order of the primers was map 11, map 2, map 4, map 7, map 8, map 9, map 10, map 12, map 13, map 14, map 15, map 18, map 17 and map 16. Primers used for sequencing the aminoglycoside resistance genes *aac (6′)-aph (2″), ant(4)-Ia, and aph(3)-IIIa*, were as described previously^17^.

#### PCR confirmation

PCRs were performed using the following parameters: initial denaturation at 95 °C for 5 min, followed by 30 cycles of denaturation at 94 °C for 30 s, annealing at 50–60 °C for 30 s, extension at 72 °C for 1 min, and a final extension at 72 °C for 5 min. Sequencing was performed by BGI Tech (Shenzhen, China), and sequences were compared with the genome of MRSA N315.

#### Reverse-transcription quantitative PCR (qPCR)

To gain some insights into expression of IS431, reverse-transcription qPCRwas performed. RNA was extracted by using 100 μL lysozyme solution (25 mg/mL lysozyme, Sigma) and Trizol (TaKaRa). The RNA was quantified by Nanodrop 2000 C, and reverse transcribed into cDNA using a PrimeScript^TM^ 1^st^ strand cDNA Synthesis Kit (TaKaRa), following manufacturer’s instructions. Real-time PCR was performed using the IS431 primer in an IQ^TM^ 5 multicolor Real-Time PCR Detection System (BIO-RAD, USA). Each sample was assayed in triplicate. The primer pairs used were *S. aureus*_16 s F: 5′-GTGTCGTGAGATGTTGGGTT-3′ and *S. aureus*_16 s R: 5′-GTGTCGTGAGATGTTGGGTT-3′, *IS431*-F: 5′-TTAATATGACGGTGATCTTGC-3′ and *IS431*-R: 5′-GCCATTGATGCAGAGGGAC-3′. Amplification was carried out with an IQ^TM^ 5 multicolor Real-Time PCR Detection System (BIO-RAD, USA) under the following conditions: 95 °C for 2 min, followed by 40 cycles of 95 °C for 15 s, 60 °C for 60 s, and 65 °C for 60 s. Amplification data were collected and analyzed.

#### Whole-genome sequencing (WGS) and data analysis

DNA libraries were prepared using the TruSeq LT kit (Illumina, San Diego, CA, USA) according to the manufacturer’s instructions and then run on a Miseq (Illumina) for generating paired-end 300-bp reads. Briefly, *de novo* assembly was performed using CLC Genomics Workbench v8.0.5 (CLC bio A/S, Aarhus, Denmark) after quality trimming with optimal word sizes based on the maximum N50 value. Contigs were oriented and ordered using Mauve^16^ against the reference N315 chromosome. *In-silico*MLST was performed by submitting the assembled contigs to CGE server (https://cge.cbs.dtu.dk/). Syntany analysis was performed via ACT^18^. Reads were mapped to the chromosome of N315 by CLC Genomics Workbench v6.05 with default settings. Candidate SNPs were called by the algorithm basic variant detection of CLC Genomics Workbench. To acquire reliable SNPs, SNPs were filtered as previously described^19^.

#### Accession numbers

This whole-genome shotgun project has been deposited in NCBI under BioProject PRJNA328893. The GenBank accession numbers of the isolates analyzed in this study are MBTB00000000 (3503-1), MBTC00000000 (3503VR10), MBTD00000000 (4126-1), MBTE00000000 (4126VR10).

## Additional Information

**How to cite this article:** Wang, A. *et al*. A potential role of transposon IS431 in the loss of *mecA* gene. *Sci. Rep.* **7**, 41237; doi: 10.1038/srep41237 (2017).

**Publisher's note:** Springer Nature remains neutral with regard to jurisdictional claims in published maps and institutional affiliations.

## Supplementary Material

Supplementary Information

## Figures and Tables

**Table 1:** *Staphylococcus aureus* strains used in this study.

Strain	Characteristic(s)	Description	MIC(μg/ml)			References
			Vm	Ox	Gm	
3503-1	Ox^r^Vm^S^, ST5-MRSA-II	Clinical isolate from hospital -acquired pneumonia (HAP) patient	2	512	2	This study
3503V6	Ox^r^Vm^I^	3503 passaged to Vm^i^for 20 days	6	8	2	This study
3503V10	Ox^s^Vm^I^	3503 passaged to Vm^i^for 45 days	10	1	4	This study
4126-1	Ox^r^Vm^S^, ST5-MRSA-II Same pulsotype as 3503-1	Clinical isolate from HAP patient	2	512	≥16	This study
4126VR10	Ox^r^Vm^i^	4126 passaged to Vm^i^	10	128	≥16	This study
3347-1	Ox^r^Vm^S^, ST239-MRSA-III	Clinical isolate from HAP patient	1	256	≥16	This study
3347-2	Ox^r^Vm^i^	3347 passaged to Vm^i^	10	16	≥16	This study
3564-1	Ox^r^Vm^S^, ST239-MRSA-III	Clinical isolate from HAP patient	2	256	≥16	This study
3564-2	Ox^r^Vm^i^	3564 passaged to Vm^i^	8	8	≥16	This study
4180-1	Ox^r^Vm^S^, untypedSSCmec	Clinical isolate from HAP patient	2	512	≥16	This study
4180-2	Ox^r^Vm^i^	4180 passaged to Vm^i^	10	128	≥16	This study
N315	Ox^s^Vm^s^	MRSA carrying type II SCC*mec,*	0.5	64	ND	ref. 20
mu50	Ox^s^Vm^i^	Clinical VISA isolate, Japan, 1997	4	256	ND	ref. 20

NOTE: Abbreviations: Ox, oxacillin; Vm, vancomycin; Gm, Gentamicin; r, resistant; s, sensitive; i, intermediate; ND, not determined.

**Table 2:** Primers used in this study.

Primer	Sequence (5′–>3′)	Description	Origin
Map2	F-GCATGCTGCTTGCCTTAGG	Maps SCC*mec* deletion	ref. 5
	R-CACACAGCCAAAGCAATCAGC	Maps SCC*mec* deletion	
Map4	F-GGTTTCATGTTTGTGCTTCAGG	Maps SCC*mec* deletion	ref. 5
	R-CACGATACAAATCAAAAAAAGGTTGG	Maps SCC*mec* deletion	
Map7	F-GTTTCAGACTTTAGCGAGGAATGG	Maps SCC*mec* deletion	ref. 5
	R-CTATGTTGTATTTATCTTCGATAATGG	Maps SCC*mec* deletion	
Map8	F-GTGTTGCATTTGGTAGCC	Maps SCC*mec* deletion	ref. 5
	R-CGATGAGTTAAGAGCACGTATC	Maps SCC*mec* deletion	
Map9	F-CCGTTCGTTATAAATACTGCC	Maps SCC*m*ec deletion	ref. 5
	R-CATGGAAAGTACATATAAAAAAAGAGG	Maps SCC*mec* deletion	
Map10	F-TATACTCAGGTGTAGGAATG	3503VR6 SCC*mec* deletion site connection	This study
	R-CGCTAAGATATCCTTCTAGT	Maps SCC*mec* deletion	
Map11	F-TGGTTCCTCAATACTAGAAG	Maps SCC*mec* deletion	This study
	R-GATAAGACACTCAAGGAAGT	3503VR6 and 3503VR10 SCC*mec*deletion site connection	
Map12	F-TAGTAACTTGGCGCTCATCA	Maps SCC*mec* deletion	This study
	R-GAACAAGGTGGGATTTCATG	Maps SCC*mec* deletion	
Map13	F-TGCTCTTAGTGTTCAACAGA	Maps SCC*mec* deletion	This study
	R-GTTCATAGCGCTGATATGAC	Maps SCC*mec* deletion	
Map14	F-ATCATTGGCCTTTTCATTGG	Maps SCC*mec*deletion	This study
	R-CGATACTACATGCAGAAGTT	Maps SCC*mec* deletion	
Map15	F-CGCGTAAGCTTTCTTCATTC	Maps SCC*mec* deletion	This study
	R-GGTACTTAAGTGGGTTAGTT	Maps SCC*mec* deletion	
Map17	F-CATCATTAGGAATCGCGAAT	Maps SCC*mec* deletion	This study
	R-TGTGTATTACAGACCTGGTT	Maps SCC*mec* deletion	
Map18	F-TCCACCTTTATACTTCGTCA	Maps SCC*mec* deletion	This study
	R-ATGTCGATGTGACTAAGGAC	Maps SCC*mec* deletion	
Map16	F-GTATCCGCTTGTTAAATGTG	Maps SCC*mec* deletion	This study
	R-GACGAGTGAACTTAACCAAT	Maps SCC*mec* deletion	
16RFAN	ATTGGTTAAGTTCACTCGTC	3503VR10 SCC*mec* for sequencing deletion site connection	This study
aac(6)-aph(2)	F-CCAAGAGCAATAAGGGCATACC		ref. 18
	R-CACACTATCATAACCATCACCG		
ant(4)-Ia	F-CTGCTAAATCGGTAGAAGC		ref. 18
	R-CAGACCAATCAACATGGCACC		
aph(3)-IIIa	F-CTGATCGAAAAATACCGCTGC		ref. 18
	R-TCATACTCTTCCGAGCAAAGG		

**Table 3:** SNPs detected between strains 3501 and 3503VR10.

SNP	3501	3503VR10	Annotation	Amino acid change
1	T	C	Iron-sulfur cluster regulator IscR	p.Val18Ala
2	G	A	Chromosome partition protein smc	/
3	C	T	Dihydroxyacetone kinase family protein	p.Asp82Asn
4	C	T	/	/
5	A	G	DNA-directed RNA polymerase beta subunit (EC 2.7.7.6)	p.Thr862Ala
6	C	T	/	/
7	C	T	/	/
8	T	C	/	/
9	C	T	/	/
10	T	C	/	/
11	A	C	/	/
12	T	C	/	/
13	C	T	CTP synthase (EC 6.3.4.2)	p.Arg93Lys
14	T	C	Teichoic acid export ATP-binding protein TagH (EC 3.6.3.40)	p.Asp85Gly

**Table 4:** SNPs detected between 4126-1 and 4126VR10.

SNP	4216-1	4216V10	Annotation	Amino-acid change
1	C	G	DHH family phosphoesterase	p.Thr526Arg
2	G	T	ornithine aminotransferase 1 (*rocD1*)	p.Leu297Ile
3	G	A	/	/
4	C	T	GGDEF domain-containing protein (diguanylate cyclase activity)	p.Gly314Ser
5	C	T	/	/
6	C	T	/	/
7	G	C	6-phosphogluconate dehydrogenase (*gndA*)	p.Pro305Arg
8	G	T	mannosyl-glycoprotein endo-beta-N-acetylglucosaminidase (*gad*)	p.Tyr269*
9	A	T	/	/
10	T	A	UDP-N-acetylglucosamine 1-carboxyvinyltransferase (*murA*)	p.Arg282*
11	G	A	DNA binding protein	p.Val25Ile

**Table 5:** Primers used for classification of SCC*me*c.

Gene	Primer sequences(5′–3′)	Product size(bp)
Scc*mec* Typing		
Type I	F-CCTTTAAAGAGTGTCGTTACAGG	613
	R-CTTCTCTCATAGTATGACGTCC	
Type II	F-CGTTGAAGATGATGAAGCG	398
	R-CGAAATCAATGGTTAATGGACC	
Type III	F-CCATATTGTGTACGATGCG	280
	R-CCTTAGTTGTCGTAACAGATCG	
Type IVa	F-GCCTTATTCGAAGAAACCG	776
	R-CTACTCTTCTGAAAAGCGTCG	
Type IVb	F-TCTGGAATTACTTCAGCTGC	493
	R-AAACAATATTGCTCTCCTC	
Type IVc	F-ACAATATTTGTATTATCGGAGAGC	200
	R-TTGGTATGAGGTATTGCTGG	
Type IVd	F-CTCAAAATACGGACCCCAATACA	881
	R-TGCTCCAGTAATTGCTAAAG	
Type V	F-GAACATTGTTACTTAAATGAGCG	325
	R-TGAAAGTTGTACCCTTGACACC	
mecA	F-GTGAAGATATACCAAGTGATT	147
	R-ATGCGCTATAGATTGAAAGGAT	
