# Supplementary material for: A potential role of transposon IS431 in the loss of mecA gene
Source: Sci Rep. 2017 Jan 25;7:41237. doi: 10.1038/srep41237 (PMC5264636; doi:10.1038/srep41237)
Supplement: Supplementary Information [file srep41237-s1.pdf]

These are the supplementary materials of our manuscript (SREP-16-01706A) entitled "A potential role of transposon IS431in the loss of *mecA* gene". The authors of our manuscript are Aihua Wang[1#], Kai Zhou[2 #], Yang Liu[3#], Liang Yang[3],Qin Zhang[4], Jing Guan[1], Nanshan Zhong[1],Chao Zhuo[1*].

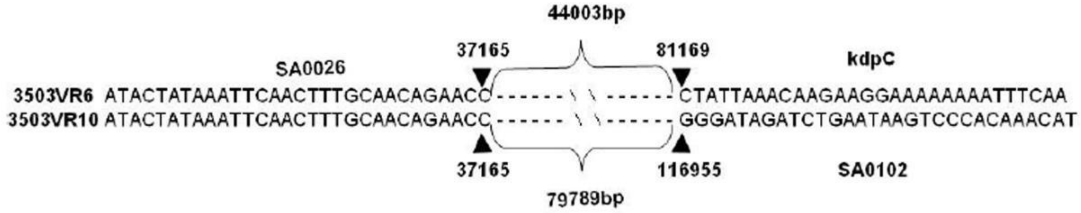

Figure S1. SCC*mec* deletion site in 3503VR6 and 3503VR10

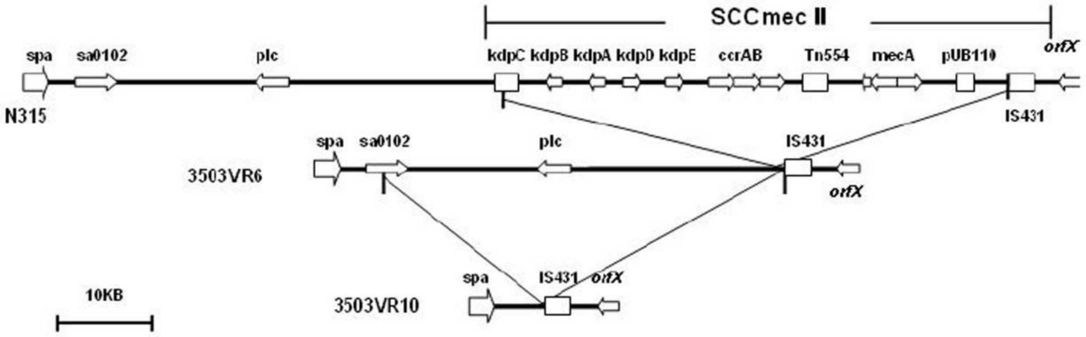

Figure S2. Schematic diagram showing the deletion regions of strains 3503VR6 and 3503VR10
